# COVID-19 and Psychological Health of Female Saudi Arabian Population: A Cross-Sectional Study

**DOI:** 10.3390/healthcare8040542

**Published:** 2020-12-09

**Authors:** Syed Mohammed Basheeruddin Asdaq, Sara Abdulrahman Alajlan, Yahya Mohzari, Mohammed Asad, Ahmad Alamer, Ahmed A. Alrashed, Naira Nayeem, Sreeharsha Nagaraja

**Affiliations:** 1 College of Pharmacy, AlMaarefa University, Riyadh 13713, Saudi Arabia; emadfaiqa@gmail.com; 2 Pharmacy Department, Clinical Pharmacy Section, King Saud Medical City, Riyadh 12746, Saudi Arabia; yali2016@hotmail.com; 3 Department of Clinical Laboratory Science, College of Applied Medical Sciences, Shaqra University, Shaqra 11911, Saudi Arabia; basheer_1@rediffmail.com; 4 Department of Pharmacy Practice and Science, College of Pharmacy, University of Arizona, Tucson, AZ 85721, USA; mhospital1920@gmail.com; 5 Department of Clinical Pharmacy, Prince Sattam Bin Abdulaziz University, Alkharj 11942, Saudi Arabia; 6 Pharmaceutical Service Department, Main Hospital, King Fahad Medical City, Riyadh 11564, Saudi Arabia; alarashed@gmail.com; 7 Department of Pharmaceutical Chemistry, Northern Border University, Arar 73214, Saudi Arabia; farhana.basheer13@gmail.com; 8 Department of Pharmaceutical Sciences, College of Clinical Pharmacy, King Faisal University, Al-Ahsa 31982, Saudi Arabia; sharsha@kfu.edu.sa; 9 Department of Pharmaceutics, Vidya Siri College of Pharmacy, Off Sarjapura Road, Bengaluru 560 035, Karnataka, India

**Keywords:** COVID-19, mental health, Impact of event scale, negative attitude, Saudi Arabian females

## Abstract

The influence of the COVID-19 pandemic is unprecedented on physical and mental health. This study aimed to determine the impact of the COVID-19 event on mental health among Saudi Arabian females of Riyadh by a cross-sectional study design. The samples of the study were recruited using convenience and snowball sampling methods. The questionnaire is composed of items related to sociodemographic profile, general mental status, negative attitude scale, impact of event (COVID-19 pandemic) scale (R) and negative health impact. The data obtained were analyzed using multivariate regression analysis. Out of the 797 samples (34.58 ± 12.89 years), 457 (57.34%) belonged to an age group of ≥25 years. The average BMI of the participants was 26.73 (kg/m^2^). Significantly (*p* = 0.000), a large proportion of the participants were overweight and unemployed. Age group (>25 years) have more odds for abnormal mental status (OR; 1.592), development of negative attitudes (OR; 1.986), the intense impact of COVID-19 events (OR; 1.444) and susceptibility to attain negative health impacts (OR; 1.574). High body weight is another risk factor for altered mental status, negative attitude and developing impact of COVID-19 quickly. Overall, the COVID-19 pandemic was directly associated with stress (53%), anxiety (63%) and depression (44%) in our sample population. There is an urgent need for psychological counseling for the distressed population.

## 1. Introduction

The unprecedented situation of COVID-19 presents a remarkable threat to the health of the general public. The presence of this highly contagious disease with the unpredictable extent of morbidity and mortality rates has an impact on almost all aspects of daily life [1]. During this difficult pandemic time, it is likely that mental health issues may get exacerbated due to perceive fear, worry, and stress because of uncertainty or factors over which humans have no control. More than one-third of the adults from the United States have shown symptoms of anxiety or depression during the pandemic, in contrast, to a figure of one in ten; from January to June 2019 [2]. In addition to the threat of getting infected with the virus, alterations in daily activities like restricted movements and strict maintenance of social distancing in several countries, new normal of work from home, partial or complete loss of a job, virtual classes for children, and avoidance of contact with friends and relatives, are considered as major contributors for altered mental functions during this crisis [3]. Concerning the susceptibility for psychological disturbance, the impact of a major epidemic is directly associated with the ability of a person to cope up with the situation. It is not wrong to say that almost the entire population has experienced some degree of mental distress during this difficult time, but the significant impact is seen only in vulnerable individuals. Particularly those people who got infected with the disease, those at high risk, such as the geriatric population, immunocompromised individuals, those living or receiving care in congregate settings, and people with preexisting psychiatric or substance abuse problems, possess an enhanced risk for abnormal psychosocial outcomes [4]. On top of that, long-term lockdown due to pandemic also results in limited access to healthcare that invariably results in mental health issues [5]. Moreover, females are more likely to develop psychological burden than males [6]. Additionally, a significant impact on mental health is also reported in people who have limited resources to use virtual social and health awareness services [7]. In addition, it is necessary to understand that all psychological illness and socialization issues are not necessarily can be termed as diseases; most of them temporary reactions to abnormal situations. However, it must be addressed in time to prevent the occurrence of its consequences.

Recently published articles emphatically describe the implication of COVID-19 on the mental health of health care professionals [8] as they are the front line warriors for this virus, and also several reports published on the implication of this situation on the educational system [9]. The reports describing the role of the pandemic on the mental status of the community [8,10] are also published elsewhere. However, there is a scarcity of data on the direct impact of COVID-19 on the mental status of the female population of Saudi Arabia. Hence, this study aimed to determine the impact of the COVID-19 event on mental health and its related lifestyle habits among Saudi Arabian females of Riyadh.

## 2. Materials and Methods

### 2.1. Sampling

This is a cross-sectional study carried out from March 2020 until the end of August 2020. The study period coincides with the progression of COVID-19 cases in Saudi Arabia. The number of new cases has been increasing many-fold, ranging from 100 odd cases in March 2020 up to 4919 cases on 17 July 2020. The number of deaths was in single digits during March that went to a peak of 58 deaths on 5 July 2020. However, due to stringent regulations of Saudi Arabian authorities, the spread of the pandemic was well controlled, with the number of new recoveries per day was almost similar to or higher than new reported COVID-19 cases. Additionally, the media of Saudi Arabia was helping the governmental authorities in restricting the spread of rumors. Overall, the situation was alarming but well under control. Ethical approval to conduct this study was obtained from the Research Committee of College of Pharmacy, AlMaarefa University, Riyadh, with approval number (MCST (AU)-COP 2001/RC). Only female adults (aged ≥ 18 years) of Saudi Arabian nationality who provided verbal informed consent and reside in Riyadh (Saudi Arabia) were recruited in the study using convenience and snowball sampling methods. The content validity and reliability check of the questionnaire were done by Cronbach’s alpha. The validated questionnaire was translated into the Arabic language, and linguistic validation was done to validate the conceptual translation of the questionnaire with the help of two qualified bilingual (English and Arabic languages) health science researchers. Pretest of the Arabic version of the questionnaire was conducted to assess the clarity of the questionnaire, suitability to the participants, the time required to complete the questionnaire and to know the possible obstacles.

The sample size was calculated (http://sampsize.sourceforge.net) based on the 28% prevalence of psychological illness in the female population of Saudi Arabia reported in one of the studies [11] with a 5% as a precision percentage and a 95% confidence level. The required sample size was 310 for the infinite population.

### 2.2. Study Questionnaire

The questionnaire used in this study had five major sections: (1) sociodemographic information such as age, education level, employment status, height and weight (to calculate BMI); (2) General mental status; (3) Negative attitude scale; (4) Impact of event (COVID-19 pandemic) Scale (R) and (5) Negative health impact.

### 2.3. General Mental Status (GMH)

The validated Arabic version of the 8-item section with Cronbach’s alpha of 0.70 was used to explore the basic mental profile of the participants during this pandemic. The items included were perceived depression status during the pandemic, medication used to control psychological burden, soliciting psychologist help, participation in mental health program during the pandemic, joining audio broadcast of mental health issues, initiating stress reliever exercise, nightmares during the pandemic and developing bad habits. The response for each question was scored 0 (no) and 1 (yes). A cutoff of ≥3 was used to reflect abnormal mental status.

### 2.4. Negative Attitude Scale (NA)

There were seven questions included in this section that were focused on elucidating the perceived negative attitude developed during the pandemic. These questions had a Cronbach’s alpha of 0.92. The questions were meant to evaluate the interest level, social life, feelings of frustration and despair, tension level, ability to focus and mood status. The responses for each question were scored 0 (never), 1 (rare), 2 (sometimes), 3 (most of the time) and 4 (always), with a lower score indicating a low negative attitude. A cutoff of ≥ 8 was used to reflect the presence of a negative attitude.

### 2.5. Impact of Event (COVID-19 Pandemic) Scale (R) (IES)

Daniel Weiss and Charles Marmar developed the first draft of the impact of event scale (IES) in 1997 [12] to parallel the DSM-IV criteria, subscale with hyperarousal items were included into IES and renamed as IES-R [13]. This scale comprises of total 22 items measured on a 5-point Likert scale rated from 0 to 4 (0 = not at all, 1 = a little, 2 = moderately, 3 = quite a bit and 4 = extremely) based on the extent to which 22 items described in the scale has caused distress to the participants in the last 7 days with reference to COVID-19. The consistency of the items was found to have Cronbach’s alpha of 0.94. The cutoff score reported in earlier literature ranges from 25 to 40, with a score of more than cutoff indicate a person at high risk for psychological problems [14]. Since this study was done during the time of the event, the investigators decided on a mean score of 35 as the cutoff point to validate the impact of COVID-19 in the participants. Intrusion (items 1, 2, 3, 6, 9, 14, 16 and 20), avoidance (items 5, 7, 8, 11, 12, 13, 17 and 22) and hyperarousal (items 4, 10, 15, 18, 19, 21) are three subscales to this questionnaire.

### 2.6. Negative Mental Health Impact (NHI)

Participants were asked to share their opinion on the six validated questions about negative mental health impacts of the pandemic compared to the pre-pandemic period; these questions had a Cronbach’s alpha of 0.88 [15]. The stress of work, financial burden, stress from home, fear due to the COVID-19 pandemic, apprehension due to the COVID-19 pandemic, and helpless feelings due to the COVID-19 pandemic were tested in this section. The categorical responses recorded were either yes (score 0) or no (Score 1). A total score of ≥3 was termed as the cutoff score for considering the presence of negative health impact due to pandemic.

### 2.7. Statistical Analysis

The data collected was entered into SPSS IBM statistical package (version 25, IBM, Armonk, NY, USA). All results of quantitative variables were reported either as mean or frequency (percentage) (%). A chi-squared test was employed to assess if there was a significant association between categorical variables. Risk estimates of the sociodemographic factors (age, educational level and BMI) on GMH, NA, IES and NHI were determined and expressed as an odds ratio. Finally, multivariate linear regression analysis was done to assess the difference in dependent and independent variables, including age, educational level, and body weight (BMI). A *p*-value of less than 0.05 was considered significant.

## 3. Results

### 3.1. Characteristics of the Participants

Of the 1191 responses received for our questionnaire, 216 responses were excluded from our study due to incomplete information on the sociodemographic profile. Out of the remaining 797 samples (average age 34.58 years), 457 (57.34%) belonged to an age group of >25 years, and 340 (42.65%) were from the 18–25 years age group. Only 30% of the respondents were secondary school qualified, while the majority of them are better educated (69%). Most of the participants (64%) of this study were non-employed or students, whereas 36% of them were working. Concerning the status of body weight, a higher proportion of the included samples were overweight (66%), with only 34% representing normal weight. The average BMI of the participants was 26.73 (kg/m^2^). Significantly (*p* = 0.000), a high percentage of the surveyors in the higher age group were overweight and unemployed (Table 1).

### 3.2. General Mental Status, Negative Attitude, IES, Negative Health Impact by Age

The overall mean general mental status score of 1.5 ± 0.059 (mean ± SEM) was noted among the participants with a significantly (*p* = 0.001) high level of abnormal mental status in a higher age group (Odds ratio, 1.304) compared to lower to age group (Table 2). The average score for negative attitude was 7.79 ± 0.239, with a risk estimate of 1.304 for the higher age group. There was no association of age on the impact of event scale (IES on COVID-19) with an overall average of 20.83 ± 0.569 among the participants. However, the age group of ≥25 years had a relatively bigger risk estimate (1.249) for the IES score. Additionally, a significant (*p* = 0.001) link was found between negative health impact and age of the participants, with an overall mean score of 2.41 ± 0.062. The overall average of IES sub-scale intrusion, avoidance and hyperarousal were 7.55 ± 0.21, 7.05 ± 0.22 and 6.22 ± 0.5, respectively.

### 3.3. General Mental Status, Negative Attitude, IES, Negative Health Impact by BMI

As shown in Table 3, overweight participants of this study had significantly abnormal mental status (*p* = 0.000), higher negative attitude (*p* = 0.001) and increase in the IES score (impact of COVID-19 event) (*p* = 0.000). However, there was no significant association between negative health impact and bodyweight. Similarly, overweight subjects of this study have shown a relatively higher risk for abnormal mental status (1.279), negative attitude (1.189), the impact of COVID-19 (1.249) and negative health impact (1.096).

### 3.4. General Mental Status, Negative Attitude, IES, Negative Health Impact by Educational Level

There was no significant association noted when we compared education level with changes in the general mental status, development of negative attitude due to COVID, the impact of the COVID-19 on their general lifestyle and overall negative impact. On the contrary, participants with low educational level had a comparatively higher risk estimate for abnormal mental status (1.057), negative attitude (1.171) and negative health impact (1.019) (Table 4).

### 3.5. Multiple Linear Regression Analysis

Multiple linear regression analysis was done to find the impact of three categorical independent variables, age, educational level and BMI, on four dependent outcomes, namely, general mental status, negative attitude, the impact of COVID-19 event and negative health impact. Table 5 shows age as a significant predictor for abnormal general mental status, development of negative attitude, eliciting impact of COVID-19 event and susceptibility to meet negative health impact. In addition to this, high bodyweight is another reason for altered mental status, negative attitude and developing impact of COVID-19 quickly. Overall, differences in the educational level have not shown any mental health impact.

## 4. Discussion

The difficult situation humanity is going through since the outbreak and declaration of the COVID-19 pandemic is unprecedented and unimaginable. The pandemic has adversely affected all walks of life, and every person on this earth has a direct or indirect impact. COVID-19 is a physical health problem, but it has the potential to cause a major mental health crisis if adequate and necessary steps are not taken in time. The World Health Organization recognized the implication of the COVID-19 pandemic on mental health and psychological functions and released a list of considerations to the public, health care workers, team leaders, and people under isolation and all other susceptible people to cut its impact [16]. Further, the United Nations proposed their recommendations to neutralize and combat the poor mental health outcomes by providing access to mental healthcare through creative means utilizing all other available and possible resources, especially across high-risk populations [17]. In addition to this, the Centers for Disease Control and Prevention (CDC) has shared measures and methods to overcome stress [18].

Although COVID-19 may produce altered mental health in any person, the section of the community vulnerable to mental alteration may get affected quickly. The mental health of all people of society is critical for the best functioning of the community. The well-being of the female population is a necessary element for the overall welfare of the system. Generally, women are more vulnerable to negative life events than men are, especially those without social support. A study carried out in Egypt reported a high prevalence of depression and anxiety in girls that are almost double that in boys [19]. In addition to this, there are several studies available in affirmation of the high incidence of mental abnormalities and quicker impact of events in females than men. Hence, the idea of this research to explain the impact of the ongoing pandemic on this vulnerable population of Riyadh, Saudi Arabia.

Overall, the mental status of the participant indicated mild alteration; however, around 44% of the participants acknowledge perceived depression due to the COVID-19 outbreak. The higher incidence of perceived depression in Saudi Arabian women is in accordance with the current global scenario [20]. People are continuously under fear of contracting infection, dying, and losing family members. Frequent misinformation through social media and other communication channels and nightmares about the future are common factors for the induction of depression. Possibly there is a role of organic changes in the central nervous system during the COVID-19 outbreak [21].

The psychological burden and alteration in mental status is a common feature of traumatic events. Studies done earlier have shown the negative implication of large scale traumatic events on the mental illness in the majority of vulnerable populations [22]. Additionally, the presence of co-morbid or riskier conditions may further enhance the impact of events. The impact of event scale-revised (IES-R) is one of the suitable scales subjectively measure the traumatic event such as COVID-19, especially in the response sets of intrusion (intrusive thoughts, nightmares, intrusive feelings and imagery, dissociative-like reexperiencing), avoidance (numbing of responsiveness, avoidance of feelings, situations, and ideas), and hyperarousal (anger, irritability, hypervigilance, difficulty concentrating, heightened startle), as well as total subjective stress IES-R score. The average score of this scale in our study showed a milder impact on the majority of the respondents, with an overall average of around 21. However, higher age groups and people of excess body weight have shown greater vulnerability to the COVID-19. The outcome of this study is in accordance with other studies reported earlier [10]. It is also interesting to note that the overall impact of the event was mild; however, 57% of the respondents still expressed added stress due to the pandemic, and 63% of them also feel apprehensive due to continuous reports of the pandemic. Probably, high stress and apprehension were due to the daily report of 2000–5000 new cases from the Kingdom of Saudi Arabia during the time of this study. Our findings are also consistent with other published literature showing that having exposure to life stressors are directly associated with more depression during times of social isolation as well as at low-intensity periods [23,24,25]. In addition to the adverse impact of COVID-19 on the economy of the country, an individual’s economic status is also adversely affected due to the pandemic in many countries. However, the government of Saudi Arabia took exceptional care for the economic well-being of their citizens by facilitating full salaries and wages in both the public sector and the private sector. Hence, in our study, we did not notice any significant effect of COVID-19 associated economic status on the psychological burden of the participants.

To the best of our knowledge, our study was one of the few studies that have given insights about the extent of mental disturbance experienced by the feminine gender of the Saudi Arabia population living in the capital city, Riyadh, due to the COVID-19 pandemic. Additionally, it covered several parameters of measurement of mental status ranging from general health status to negative attitude, negative impact as well as the overall impact of COVID-19 using a reliable measuring tool.

However, it is an advantage to get to know the mental status of the female population. Having the data on the male gender would have help in comparing the extent of difference between the two sets of the population. Since most parts of this study were carried out during the lockdown phase, most of the respondents were reached through the social media link. There was no support offered to the participants on the tricky questions that need clarification that may count for understanding or interpretation bias on the part of the respondents. With a high percentage of the respondents having depression (44%), stress (53%) and apprehension (63%), it would be a good idea to do a large-scale study across different regions of the Kingdom of Saudi Arabia. Further, having data on the specific aspect of COVID-19, such as loss of a job, death of some beloved ones in the family, and others, could have given more insight into the specific issues. Additionally, as the number of cases dropping down in the Kingdom, the latest research will be needed to assess the trajectory of depression in the Saudi population and develop the potential treatment for affected populations.

## 5. Conclusions

The COVID-19 pandemic is associated with stress (53%), anxiety (63%) and depression (44%) in our sample population. Participants in the higher age group and overweight people have a high risk for alteration in mental health. Large scale study spread across different regions of Saudi Arabia, covering several types of population needed to assess the trajectory of the mental health of the Saudi population.

## Figures and Tables

**Table 1:** Sociodemographic characteristics of the participants.

Variables	All, n (%)	18–25 Years, n (%)	>25 Years, n (%)	*p* Value ^1^
Education level				
Higher qualification, n (%)	556 (69.8)	228 (67.1)	328 (71.8)	0.152
Secondary school, n (%)	241 (30.2)	112 (32.9)	129 (28.2)	
Employment status				
Employed, n (%)	287 (36)	93 (27.4)	194 (42.5)	0.000
Non employed, n (%)	371 (46.5)	113 (33.2)	258 (56.5)	
Students, n (%)	139 (17.4)	134 (39.4)	5 (1.1)	
BMI				
Normal weight, n (%)	273 (34.3)	158 (46.5)	115 (25.2)	0.000
Overweight, n (%)	524 (65.7)	182 (53.5)	342 (74.8)	

^1^ Pearson chi-squared test.

**Table 2:** General mental status, negative attitude, impact of event scale (IES), negative health impact by age.

Variables	All, n (%)	18–25 Years, n (%)	>25 Years, n (%)	*p* Value ^1^
General mental status				
Normal, n (%)	614 (77)	372 (81.4)	242 (71.2)	0.001
Abnormal, n (%)	183 (23)	85 (18.6)	98 (28.8)	
Odds ratio (risk estimate) ^2^		0.736	1.304	
Negative attitude				
Absent, n (%)	466 (58.5)	164 (48.2)	302 (66.1)	0.000
Present, n (%)	331 (41.5)	176 (51.8)	155 (33.9)	
Odds ratio (risk estimate) ^2^		0.662	1.304	
Impact of COVID-19 event				
Absent, n (%)	640 (80.3)	258 (75.9)	382 (83.6)	0.007
Present, n (%)	157 (19.7)	82 (24.1)	75 (16.4)	
Odds ratio (risk estimate) ^2^		0.772	1.249	
Negative health impact				
Absent, n (%)	446 (56)	167 (49.1)	279 (61.1)	0.001
Present, n (%)	351 (44)	173 (50.9)	178 (38.9)	
Odds ratio (risk estimate) ^2^		0.760	1.234	

^1^ Pearson chi-squared test; ^2^ risk estimate for 2 × 2 table.

**Table 3:** General mental status, negative attitude, IES, negative health impact by body weight (BMI).

Variables	All, n (%)	Normal Weight, n (%)	Overweight, n (%)	*p* Value ^1^
General mental status				
Normal, n (%)	614 (77)	189 (69.2)	425 (81.1)	0.000
Abnormal, n (%)	183 (23)	84 (30.8)	99 (18.9)	
Odds ratio (risk estimate) ^2^		0.671	1.279	
Negative attitude				
Absent, n (%)	466 (58.5)	138 (50.5)	328 (62.6)	0.001
Present, n (%)	331 (41.5)	135 (49.5)	196 (37.4)	
Odds ratio (risk estimate) ^2^		0.726	1.189	
Impact of COVID-19 event				
Absent, n (%)	640 (80.3)	202 (74)	438 (83.6)	0.001
Present, n (%)	157 (19.7)	71 (26)	86 (16.4)	
Odds ratio (risk estimate) ^2^		0.698	1.249	
Negative health impact				
Absent, n (%)	446 (56)	141 (51.6)	305 (58.2)	0.077
Present, n (%)	351 (44)	132 (48.4)	219 (41.8)	
Odds ratio (risk estimate) ^2^		0.841	1.096	

^1^ Pearson chi-squared test; ^2^ risk estimate for 2 × 2 table.

**Table 4:** General mental status, negative attitude, IES, negative health impact by educational level.

Variables	All, n (%)	Higher Qualification, n (%)	Secondary School, n (%)	*p* Value ^1^
General mental status				
Normal, n (%)	614 (77)	426 (76.6)	188 (78)	0.668
Abnormal, n (%)	183 (23)	130 (23.4)	53 (22)	
Odds ratio (risk estimate) ^2^		0.977	1.057	
Negative attitude				
Absent, n (%)	466 (58.5)	316 (56.8)	150 (62.2)	0.155
Present, n (%)	331 (41.5)	240 (43.2)	91(37.8)	
Odds ratio (risk estimate) ^2^		0.935	1.171	
Impact of COVID-19 event				
Absent, n (%)	640 (80.3)	449 (80.8)	191 (79.3)	0.624
Present, n (%)	157 (19.7)	107 (19.2)	50 (20.7)	
Odds ratio (risk estimate) ^2^		1.029	0.937	
Negative health impact				
Absent, n (%)	446 (56)	310 (55.8)	136 (56.4)	0.860
Present, n (%)	351 (44)	246 (44.2)	105 (43.6)	
Odds ratio (risk estimate) ^2^		0.92	1.019	

^1^ Pearson chi-squared test; ^2^ risk estimate for 2 × 2 table.

**Table 5:** Multiple linear regression analysis.

Scale	Variable	B	Std. Error	Beta	*p* Value
General Mental status	Constant	1.224	0.194		0.000 *
	Age	0.465	0.175	1.592	0.008 *
	Educational level	−0.080	0.189	0.923	0.672
	BMI	−0.540	0.177	0.583	0.002 *
Negative attitude	Constant	0.252	0.167		0.132
	Age	0.686	0.151	1.986	0.000 *
	Educational level	−0.253	0.162	0.777	0.120
	BMI	−0.331	0.156	0.718	0.034 *
Impact of COVID-19 event	Constant	1.330	0.201		0.000 *
	Age	0.367	0.185	1.444	0.047 *
	Educational level	0.103	0.195	1.108	0.599
	BMI	−0.505	0.187	0.603	0.007 *
Negative health impact	Constant	0.068	0.164		0.681
	Age	0.453	0.149	1.574	0.002 *
	Educational level	−0.043	0.157	0.958	0.78
	BMI	−0.160	0.155	0.852	0.302

* *p* value < 0.05 indicates significant comparison using chi-squared test.
